# Solvent free spray coated n type PEDOT PSS thin film for high performance homojunction diode

**DOI:** 10.1038/s41598-024-73971-y

**Published:** 2025-06-25

**Authors:** Mohsen Ghali, Cyril O. Ugwuoke, Ahmed Abd El-Moneim

**Affiliations:** 1 Institute of Basic and Applied Science, Egypt-Japan University of Science and Technology, Alexandria, Egypt; 2 Physics Department, Kafrelsheikh University, Kafrelsheikh, Egypt

**Keywords:** Spray coating technique, n-PEDOT:PSS, Homojunction, Seebeck coefficient, Rectification ratio, Nanoscale devices, Electronic devices, Electronic properties and materials

## Abstract

Pristine PEDOT: PSS is a p-type material with salient features. However, studies have revealed that the electrical property of pristine PEDOT: PSS can be switched to n-type by solvent treatment. Notwithstanding that this method is simple, it performs inconsistently and primarily affects the samples’ surfaces due to the migration of the solvent molecules into the polymer network. These solvents are also hazardous and unfavourable to the environment. In this work, we report solvent-free switching electrical characteristics of pristine PEDOT: PSS to n-type using a spray coating technique. The obtained n-PEDOT: PSS exhibits excellent optical and electrical characteristics. In addition, high Seebeck coefficient of – 1320.06 ± 38.42 µVK^−1^ and power factor of 4.32 ± 0.25 µWm^−1^K^−2^ were found. The obtained n-PEDOT: PSS was used in the fabrication of a homojunction diode. The I-V curve showed rectification characteristics with rectification ratio and barrier height of 17.2 and 0.047 eV, respectively, which is one of the best reported values in the literature for all PEDOT: PSS-based diode.

## Introduction

Organic electronic devices, particularly organic solar cells (OSCs), organic light-emitting diodes (OLEDs), and organic diodes, have become more and more popular in recent years thanks to their salient features such as affordability, easiness of fabrication, lightweight, and moderate transparency^1^. Nonetheless, there is a need for improvement in terms of durability and efficacy. The use of new functional materials like the hole transport layer (HTL) and the electron transport layer (ETL) and optimization of the device design are some of the improvement strategies^2^. In utilizing new functional materials, the performance of these devices depends on the material selection of ETL and HTL because they have a significant impact on the efficiency, stability and overall performance of the device^3^. Conducting polymers, e.g. Poly(3,4-ethylenedioxythiophene): poly(styrenesulfonic acid) (PEDOT: PSS), polyaniline (PANI), PPV-polymer Super Yellow (SY), etc., have been used either as the ETL or the HTL^4,5^.

Among them, PEDOT: PSS has been the most outstanding due to its unique properties, such as high transparency, thermal stability, and excellent work function^6,7^. Notwithstanding the merits, PEDOT: PSS on the other hand, has some characteristics that restrict its range of applications^8^. The most significant downside of PEDOT: PSS is its poor conductivity^9^ and hydrophilicity, which severely restricts its ability to disperse over hydrophobic photoactive material like poly (3-hexylthiophene): 1-(3-methoxycarbonyl) propyl-1-phenyl [6, 6] C61 (P3HT: PCBM) in OSCs^10^. These limitations cause low efficiency in PEDOT: PSS-based devices. Treatment with acid, Zwitarions, salts, surfactants, ionic liquids and organic solvents have all been reported to enhance the electrical properties of PEDOT: PSS^11–13^.

While pristine PEDOT: PSS is inherently a p-type material, it exhibits a tuneable electrical property that permits switching between p-type to n-type. The switching feature is facilitated by mere solvent treatment e.g., isopropanol alcohol (IPA)^14,15^. However, IPA in PEDOT: PSS solution has a favourable impact on the shape and structure of PEDOT: PSS films which enhance electrical conductivity and homogeneous film composition^16^. Sami et al.^14^ observed the material’s transition from p-type to n-type and the developed n-PEDOT: PSS was used as an ETL in hybrid solar cells. They noted a hike in the electrical conductivity up to 500%. Aboulhadeed et al.^17^ investigated the PEDOT: PSS-based homojunction diode. They observed the switching property of PEDOT: PSS at the IPA/PEDOT: PSS volume ratio of 1:1. The diode exhibited nonlinear I-V behaviour with a low rectification ratio of 3. Although this solvent treatment is simple, it performs inconsistently and primarily affects the samples’ surfaces^18^, and it is hazardous, complicated, and unfavourable to the environment^19^. While the use of IPA facilitated the electrical switching of PEDOT: PSS, other works in the literature reported on solvent post-treatment for enhancement of the electrical properties of PEDOT: PSS. For example, Gamma-butyrolactone (GBL), dimethylacetamide (DMAC), dimethyl sulfoxide (DMSO), Dimethylformamide (DMF), ethyl glycol (EG), and sulfuric acid have been used to improve the electrical properties of PEDOT: PSS^20–23^. It is well known that DMAC, DMF and sulfuric acid materials are toxic and hazardous, especially when inhaled or in contact with skin^24^. GBL is regarded as a psychoactive drug^25^. Its use in industries is constrained because it is governed as a controlled chemical. DMSO is less hazardous to both humans and the environment, but one of the significant downsides is removing it after a reaction. Thus, achieving the electrical property switching of PEDOT: PSS without solvent treatment, which has comparable features to the pre/post-treated PEDOT: PSS, is of utmost importance.

PEDOT: PSS thin film has been deposited using various techniques, e.g. drop casting, spray coating, spin coating, etc^26,27^. The use of the spray coating technique has good merits, which include cost-effectiveness, user-friendliness, and suitability for the deposition of high-purity and uniform films^28,29^. The challenge associated with the spray coating of PEDOT: PSS is that it has poor dispassion/wettability on a glass substrate. Solvent pretreatment of PEDOT: PSS prior to spay coatings has been adopted to curtail this downside^30^. For this purpose, IPA^31,32^ and DMSO^33,34^ have been widely used. However, this process is confronted with the expansion of the PEDOT: PSS polymer matrix, resulting from the migration of the solvent molecules into the polymer network^35^. This swelling arises because the solvent molecules enter the polymer network and expand the solution’s volume. Consequently, the physical properties, e.g., the electrical and mechanical properties of the PEDOT: PSS film can be altered. Therefore, these pretreatments of PEDOT: PSS could have caused the researchers not to note the electrical switching properties of PEDOT: PSS using the spray coatings technique.

In our earlier published paper^15^, we reported the solvent-free electrical property switching of PEDOT: PSS from p-type to n-type using a spray coating technique and highlighted the thermoelectric properties of n-PEDOT: PSS. This work adopted a similar approach to preparing n-type PEDOT: PSS. The obtained n-PEDOT: PSS was used to fabricate a high-performance all PEDOT: PSS p-n diode. To the best of our knowledge, this would be the first time reporting a high-performance PEDOT: PSS-based homojunction diode using n-PEDOT: PSS obtained from spray coating technique.

## Experimental procedure

### Preparation of n-type PEDOT: PSS thin film

The concentration of 1.3% wt in H_2_O of PEDOT: PSS was purchased from Sigma-Aldrich P483095-250G and it was used for this experiment without any further purification.

The glass substrates were cut in 1 cm × 1 cm dimensions. Before being sprayed with the PEDOT: PSS solution, the glass substrates were cleaned by ultrasonicating for 30 min with 2 ml of Hellmanex III solution in 40 ml of distilled water and oven-dried thereafter. The UV ozone cleaner was used to enhance the wettability of the glass substrate.

Nadetech spray coating machine (model: ND-SP 11/4), 230 V/50Hz, and power: 1000 W was used to deposit the n-PEDOT: PSS film. Usually, compressed air pressure and ultrasonic frequency are utilized to facilitate this process. It pushes the fragmented pristine PEDOT: PSS through a nozzle. This results in fine spray covering the glass substrate uniformly forming the n-PEDOT: PSS. The optimized deposition factors, e.g., air pressure, power, frequency, substrate temperature, nozzle-substrate distance, speed, distance between steps, height, and width, were set at 0.1 Bars, 3.3 W, 94,140 Hz, 25 °C, 61 mm, 2000 mm/min, 3 mm, 10 mm and 10 mm, respectively and the spray flow rate was varied between 10, 15, 20, 25, 30, and 35 ml/h.

### Mechanism of electrical switching of PEDO: PSS

**Fig. 1:**
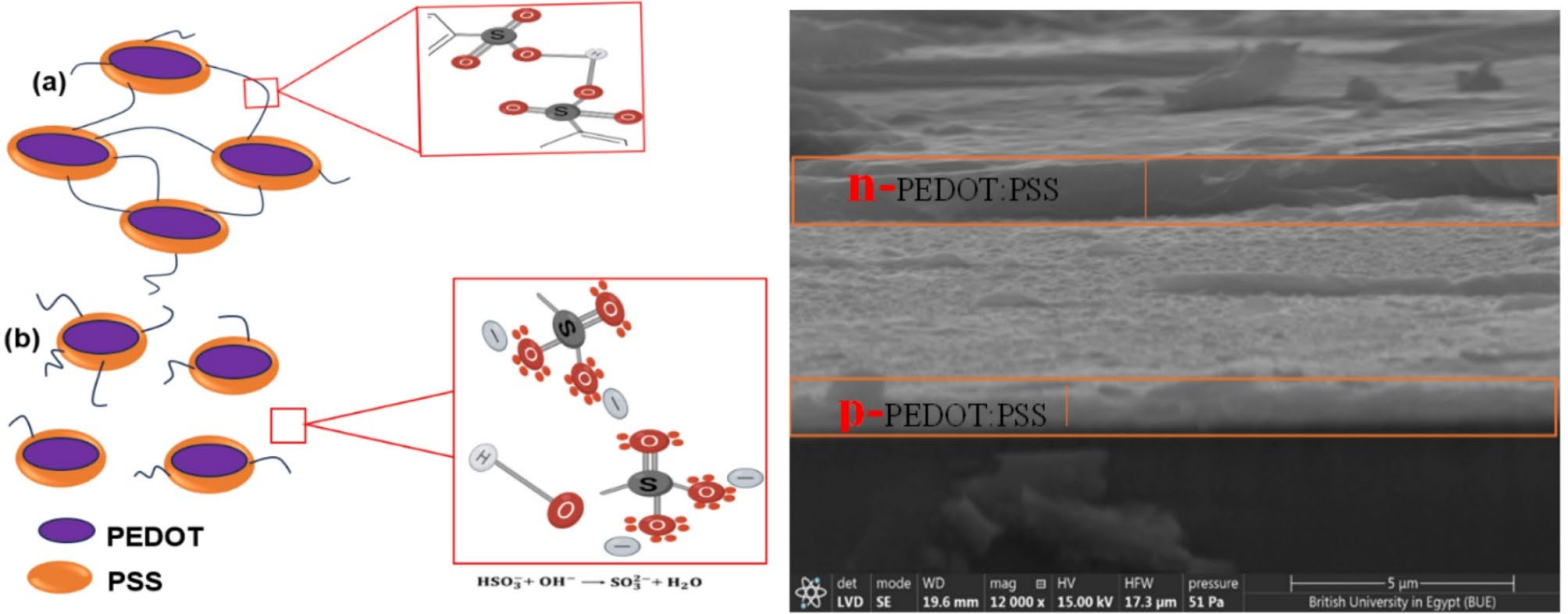
(A) The (a) bond in PEDOT: PSS and (b) bond breaking between the PEDOT and PSS (B) A cross-sectional SEM micrograph of p5:n2 diode device indicating the p and n PEDOT: PSS layers.

So far, the reported electrical switching of PEDOT: PSS has been due to IPA solvent treatment. The original organic compounds arrangement within PEDOT: PSS is shown in Fig. 1A. Figure 1A. a, shows that individual PEDOT: PSS particles are connected by HSO_3_linkages. A displacement reaction occurs when an alkanol (such as IPA) is used as an additive. The PEDOT is less soluble compared to PSS; the OH group in IPA reacts with HSO_3_ linkage in PEDOT: PSS to generate sulfite ion (SO_3_^–^) and water^13^. As shown in Fig. 1B. b, the bond breakage of HSO_3_ linkage subsequently leads to the dispersion of PEDOT: PSS particles (with fewer PSS molecules) in water. The water molecule can be evaporated by thermal annealing, leaving behind the sulfite ions on the surface of PEDOT: PSS. These sulfite ions create excess electrons in the surface of pristine PEDOT: PSS, resulting in electrical switching. This process, on the other hand, can cause a phase change^17^. The mechanism of electrical switching of PEDOT: PSS from p-type to n-type is still a subject of debate among researchers because many have observed similar phase changes in post-treated pristine PEDOT: PSS, yet it maintains a p-type material. Basically, the phase change observed from PEDOT: PSS after solvent/acid treatment could be due to the removal of PSS molecules at the surface. The electrical switching observed in this work is believed to be based on PEDOT: PSS bond rearrangement caused during the atomization process. The working principle of the spray coating technique is based on the atomization process—which involves applying the pristine PEDOT: PSS coating on a surface by fragmenting it into tiny droplet particles. This atomization is caused by high-frequency ultrasonic vibration and air pressure. The high-frequency ultrasonic vibration and air pressure may lead to cavitation and localized heating in materials. This occasionally results in the disruption of non-covalent interactions or the breaking of weaker chemical bonds^36^. However, the polymer chains within the droplets of PEDOT: PSS may align and orient due to the turbulence and shear forces produced during atomization. The electronic structure and charge transport characteristics of the conjugated polymer backbone may alter as a result of this alignment, resulting in the formation of n-type material.

### Characterizations of n-type PEDOT: PSS thin film

The properties of n-PEDOT: PSS thin film was studied by using different characterization equipment. Using an X-ray diffractometer (XRD) 6100 F from Shimadzu Company, the structural properties of the prepared n-PEDOT: PSS thin film were examined. The phase separation between PEDOT and PSS was studied by using a Hitachi AFM5500M Atomic Force Microscope (AFM). Hitachi U-3900 UV-vis spectrophotometer was used to study the optical properties of the film. The Hall Effect Measurement System with a Jandel four-probe device model RM3000, a precise magnetic field strength, and sample geometry was used to measure the electrical properties of n-PEDOT: PSS film. The four probes were fixed at the edges of the film and a current of 1 mA was inputted. The Hall voltage across the material was measured. A computer interface that was attached to the voltmeter was used for data acquisition.

In addition, the Seebeck coefficient of the obtained n-PEDOT: PSS film was measured using Seebeck coefficient measurement system. The system consists of a thermopile and Data acquisition system (Keithley 34970 A), and a computer. One end of n-PEDOT: PSS film was connected to the thermopile’s hot region (set at 40 °C) and the cold region was maintained at room temperature. The measurement was carried out within 8 min, and the data acquisition system was set to take the voltage reading every 1 s, making up to 480 voltage data points. The obtained voltage was used to calculate the Seebeck coefficient and power factor.

### Fabrication of homojunction diode

The Fluorine-doped Tin Oxide (FTO)/p-PEDOT: PSS/n- PEDOT: PSS/Cu homojunction diode was fabricated as follows: (i) drop casting technique was adopted to form the layer of p-type PEDOT: PSS. Pristine PEDOT: PSS was deposited on the FTO substrate using the drop-casting technique. The formed homogeneous film was allowed to dry at an ambient temperature. (ii) Spray coating technique (20 ml/hr spray flow rate) was used to form the layer of n-PEDOT: PSS. Namely, pristine PEDOT: PSS (without treatment) was spray coated on the layer of p-PEDOT: PSS to form n-PEDOT: PSS layer. The film was allowed to dry at an ambient temperature. (iii) The process described in (i) and (ii) were used to fabricate 10 diode devices by varying the thickness ratio of p- and n-layers to form what will be labelled as p5:n2, p4:n2, p3:n2, p2:n2, p1:n2, p5:n1, p4:n1, p3:n1, p2:n1; and p1:n1 diodes. Each layer of the diode was thermally treated at the temperature of 100 °C for 10 min (iv) Subsequently, a top electrode made of an ohmic contact with a Cu metal layer about 50 nm thick was thermally evaporated and placed over each diode’s n-PEDOT: PSS layer.

The thickness of p- and n- layers of the FTO/p-PEDOT: PSS/n-PEDOT: PSS/Cu fabricated diodes were measured using cross-sectional scanning electron microscopy (SEM). Low accelerating voltage was used to operate the SEM to reduce sample damage and improve surface contrast. Multiple measurements were obtained at various locations across the cross-section to account for possible variances. In addition, the error measurement was estimated by using standard deviation. Figure 1B shows a clear image of typical n- and p- layers of the fabricated diode, while the summary of both p- and n- layers thickness with their respective error measurements is presented in Table 1.

**Table 1:** The thickness of the diode layers.

Sample	Charge type	Thickness (µm)
p1	p-type	5.216 ± 0.045
p2	p-type	3.665 ± 0.048
p3	p-type	2.449 ± 0.056
p4	p-type	1.145 ± 0.099
p5	p-type	0.708 ± 0.010
n1	n-type	0.765 ± 0.009
n2	n-type	1.349 ± 0.094

## Results and discussion

### XRD measurement of p-PEDOT: PSS and n-PEDOT: PSS

**Fig. 2:**
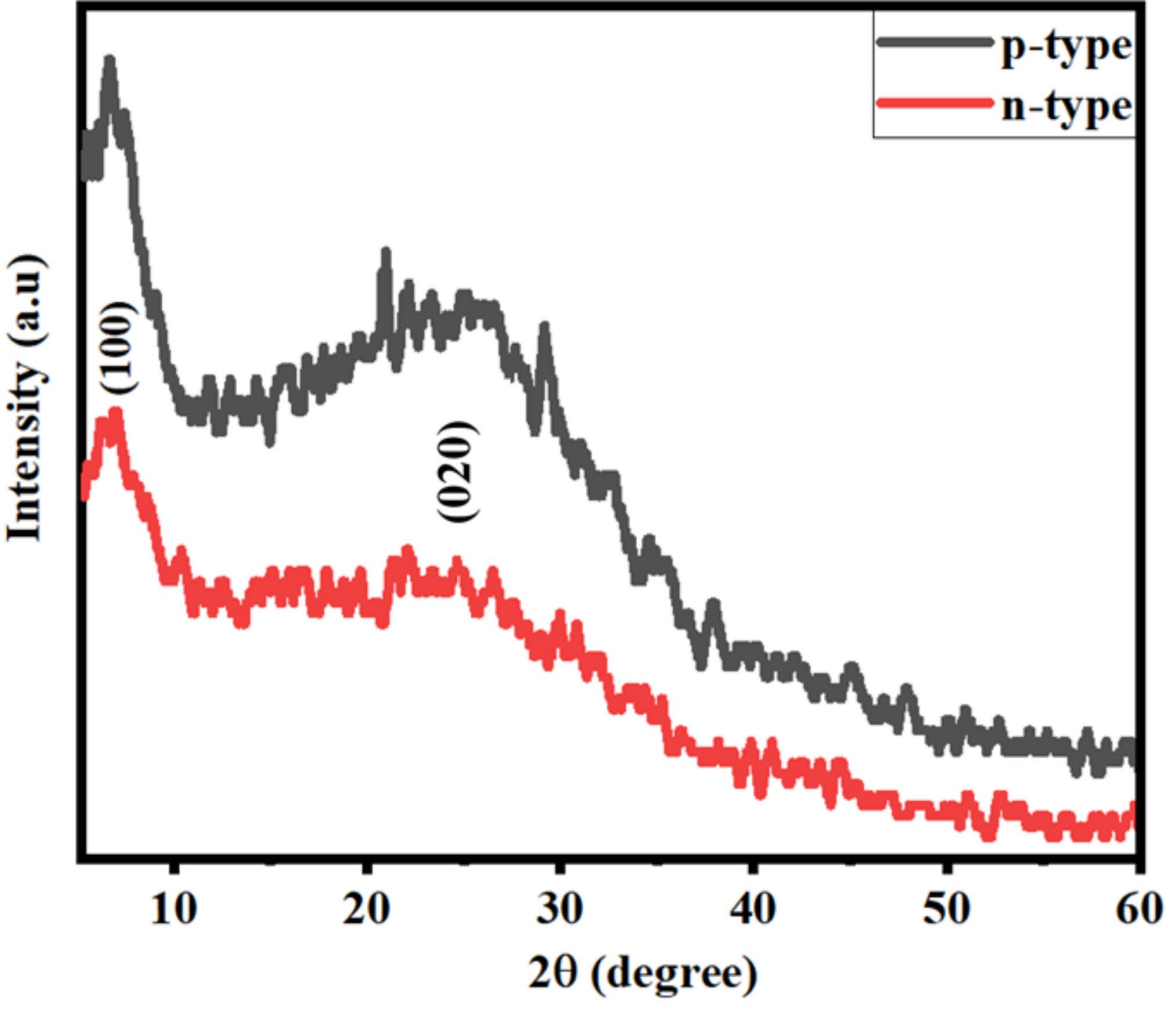
The XRD spectra of p-PEDOT: PSS and n-PEDOT: PSS layers.

The XRD spectra of p-PEDOT: PSS and n-PEDOT: PSS are presented in Fig. 2. Both samples displayed broad and weak peaks with a peak maximum of around 25.2° and 6.8° 2θ angle, respectively. Similar peaks were observed by Hu et al.^37^. The 2θ angle at 25.2° and 6.8° corresponds to (020) and (100) crystallographic planes, respectively. The broad peak could be attributed to the chaotic nature of PEDOT intermacromolecular chains, whereas the weak peak is attributed to the ring packing of PEDOT interchains across the axis of the orthorhombic structure^38^.

### **Atomic force microscopy (AFM) of p-PEDOT: PSS and n-PEDOT: PSS layer**s

**Fig. 3:**
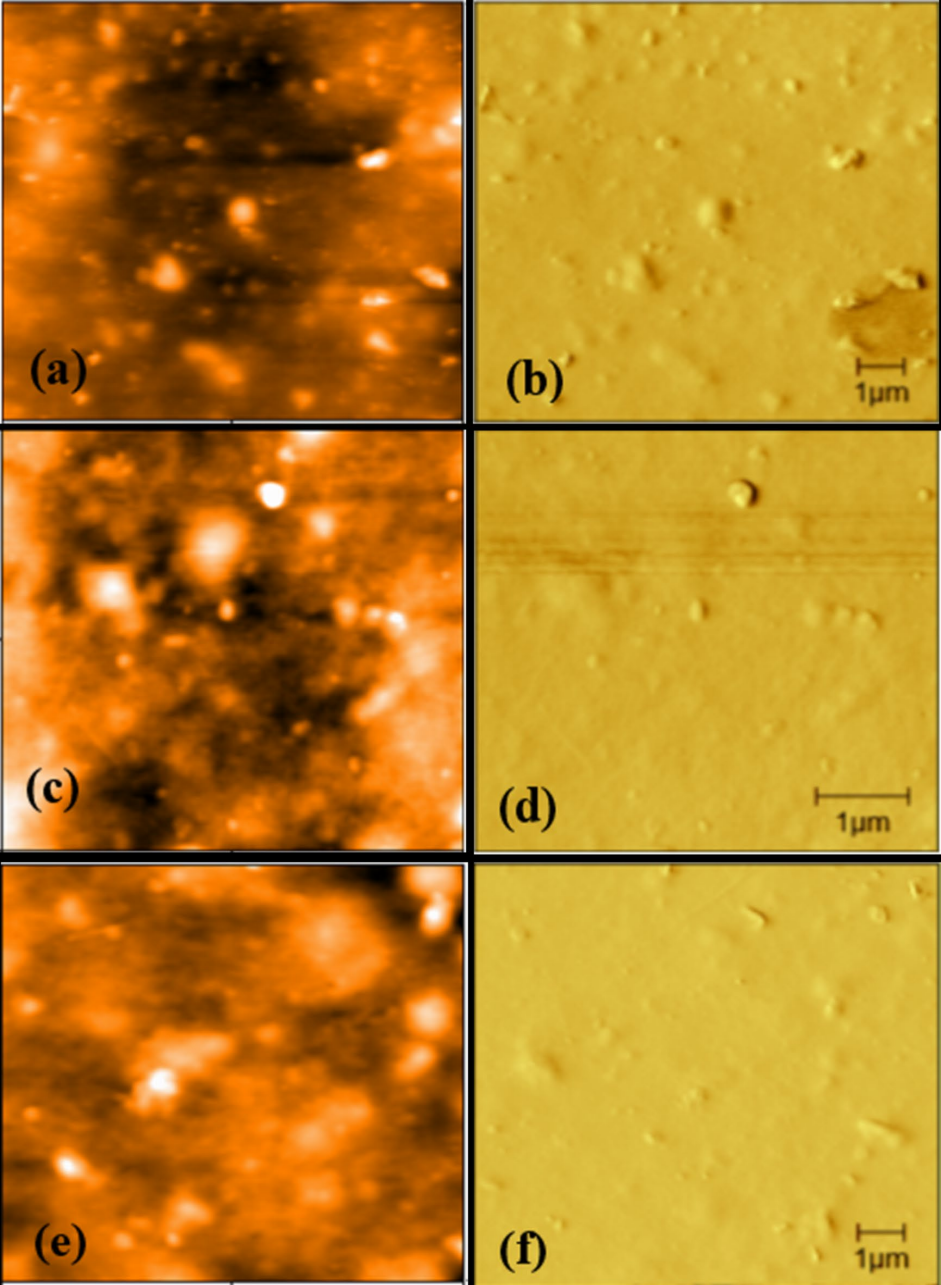
AFM micrograph (5 μm × 5 μm) of (a & b) pristine PEDOT: PSS deposited using drop casting technique (c & d) n-PEDOT: PSS deposited using spray coating technique with 10 ml/hr spray flow rate and (e & f) ) n-PEDOT: PSS deposited using spray coating technique with 20 ml/hr flow rate of spray coatings technique. *The height images are labeled a*,* c*,* and e*, whereas b, d, and f are the phase images.

The AFM micrograph (including the height and phase images) of n-PEDOT: PSS and pristine p-PEDOT: PSS films deposited using spray coating and drop casting techniques, respectively, are shown in Fig. 3. As shown, the p-PEDOT: PSS micrograph showed a phase separation between PEDOT and PSS, while n-PEDOT: PSS displayed a lesser phase separation. The obtained phase change was not pronounced due to the PSS-rich surface of both samples^39^.

The thickness and surface roughness of drop-casted film are 5.22 ± 0.045 μm and 11.68 ± 0.014 nm, respectively. Similarly, the thickness and surface roughness of n-PEDOT: PSS spray-coated film at 10 ml/hr flow rate is 0.573 ± 0.064 μm and 7.94 ± 0.055 nm respectively, while the film formed at 20 ml/hr flow rate has thickness and surface roughness of 1.349 ± 0.094 μm and 5.13 ± 0.043 nm respectively. The high value of surface roughness shown by p-PEDOT: PSS dropped-cast film means that it is less homogenous compared to n-PEDOT: PSS spray-coated films. The sample of n-PEDOT: PSS spray coated at 20 ml/hr flow rate has the least surface roughness compared to 10 ml/hr flow rate due to high film thickness. High film thickness results in low surface roughness of n-PEDOT: PSS thin film^40^.

### Optical properties

**Fig. 4:**
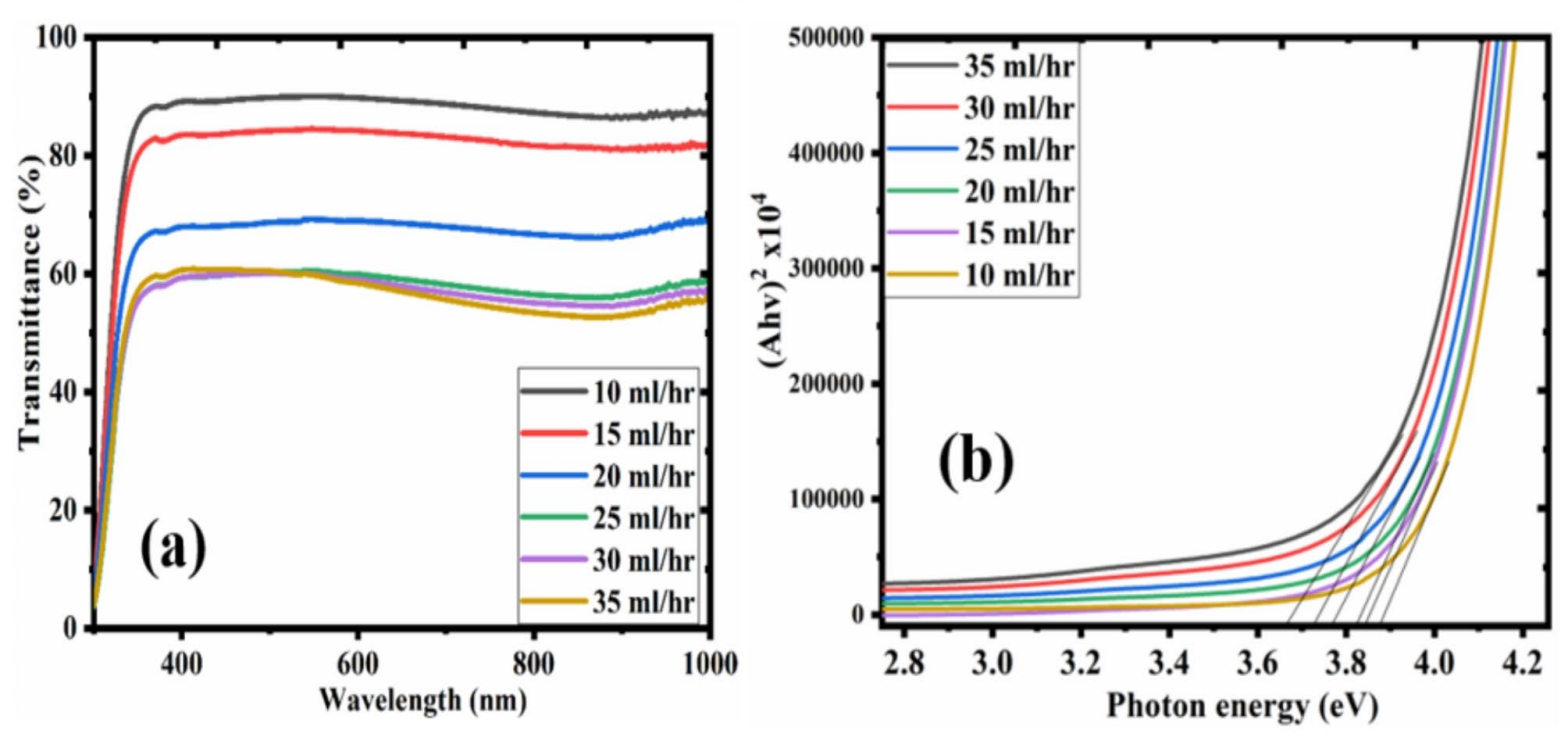
(a) Transmittance and (b) bandgap plots of n-PEDOT: PSS films at different deposition flow rates.

The optical transmittance and band gap of n-PEDOT: PSS layer is presented in Fig. 4. The films deposited at 10, 15, 20, 25, 30 and 35 ml/hr have a transmittance of 88.12, 82.34, 69.61, 59.25, 57.56, 56.03%, respectively. All the samples demonstrated excellent stability within the visible and near-infrared region, and they displayed a decrease in transmittance as the flow rate increased. The decrease in transmittance could be attributed to the decreased surface roughness and increase in film thickness as the flow rate increased.

The optical energy band gap (E_g_) of n-PEDOT: PSS was determined using the plot of $\:{\left(\propto\:hv\right)}^{2}$ against $\:hv$. The optical bandgap values for 10, 15, 20, 25, 30, 35 ml/hr flow rates are 3.78, 3.68, 3.66, 3.62, 3.59, and 3.51 eV respectively. The band gap of n-PEDOT: PSS decreases as the flow rate increases. For this reason, n-PEDOT: PSS is expected to have an improved value of electrical conductivity^41^.

### The electrical properties of n-PEDOT: PSS film

The electrical properties of n-PEDOT: PSS layers were studied using the Hall Effect Measurement system. The induced voltage is measured by introducing a magnetic field perpendicular to the current flow. Analysis of the voltage allows the study of carrier type, carrier concentration, resistivity, electrical conductivity, and sheet resistance, providing insights into the films’ electrical/electronic properties. The carrier concentration, resistivity, sheet resistance and electrical conductivity of the n-PEDOT: PSS were calculated using the following equations^32^.

1 $$\:{\uprho\:}=\frac{\pi\:t}{ln2}\left(\frac{V}{I}\right)\hspace{0.17em}=\hspace{0.17em}4.523\text{t}\frac{V}{I}$$

2 $$\:{\upsigma\:}\:=\:\frac{1}{\varvec{\rho\:}}$$

3 $$\:{N}_{d}=\frac{1}{e{R}_{h}}$$

4 $$\:{R}_{s}=\frac{{\uprho\:}}{t}$$

where $\:{N}_{d}$ is the carrier concentration, V/I is the resistance (R), R_h_ is the hall coefficient, t is the film thickness, $\:{\uprho\:}$ is the resistivity, $\:{\upsigma\:}$ is the conductivity, and Rs is the sheet resistance.

**Fig. 5:**
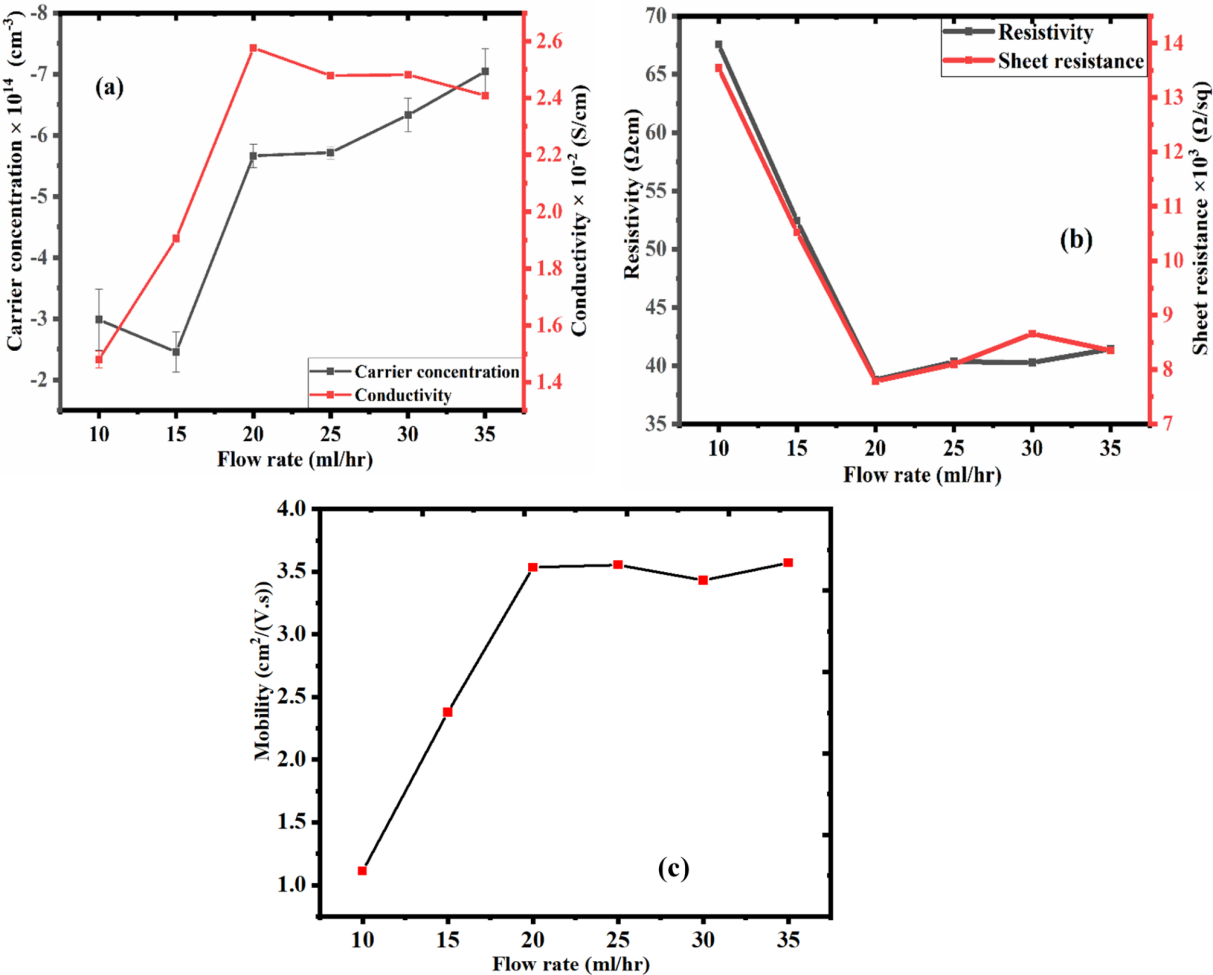
The (a) carrier concentration and conductivity (b) and resistivity and sheet resistance (c) mobility plots of n-PEDOT: PSS against the flow rate.

The effect of flow rate on carrier concentration, resistivity, electrical conductivity and sheet resistance of n-PEDOT: PSS films are presented in Fig. 5. Figure 5a shows an increase in the conductivity and carrier concentration of the deposited n-PEDOT: PSS layer as the flow rate increases. The conductivity and carrier concentration of n-PEDOT: PSS increases from 1.48 ± 0.03 × 10^−2^ to 2.48 ± 0.06 × 10^−2^ S/cm and – 2.99 ± 0.05 × 10^14^ to – 7.04 ± 0.37 × 10^14^ cm^−3^, respectively, as the flow rate increases from 10 to 35 ml/hr. This could be due to the increase in film thickness^42,43^. As the thickness of n-PEDOT: PSS increases, the total number of mobile electrons rises, which in return causes an improvement in the conductivity and carrier concentration. The excellent conductivity and carrier concentration shown by n-PEDOT: PSS makes it a candidate material for ETL in OSCs and Schottky diodes^44^.

Figure 5b shows the plots of resistance and resistivity of n-PEDOT: PSS against the flow rate. As seen, the sheet resistance and resistivity of n-PEDOT: PSS decreases progressively as the flow rate increases. The sheet resistance and resistivity of n-PEDOT: PSS ranges from 13.54 ± 0.03 × 10^3^ to 8.35 ± 0.02 × 10^3^ Ω/sq and 67.56 ± 0.01 to 41.44 ± 0.01 Ωcm, respectively for 10 to 35 ml/hr flow rate. A lower sheet resistance and resistivity typically denote more effective charge transport and improved electrical conductivity.

The Fig. 5c shows the plot of mobility against flow rate. The mobility increases range from 1.11 ± 0.005 to 3.57 ± 0.01 cm^2^/(V.s). A higher mobility suggests that charge carriers can move through the material more quickly. Increased mobility in electronic components like organic transistors or diodes results in quicker charge transport, lower resistance, and enhanced device performance^17^. Even though the sample prepared at 35 ml/hr exhibits the highest carrier concentration and mobility compared to the samples prepared at 20–30 ml/h. The lower conductivity observed on the sample prepared at 35 ml/hr could result in film defect caused by the increased film thickness.

The sample deposited at a 20 ml/hr flow rate showed the least resistivity, sheet resistance and the highest conductivity. This could be that at 20 ml/h flow rate, the spray droplet size and distribution became optimal. Smaller droplets generated at 20 ml/h flow rate lead to better atomization and dispersion of the solution, resulting in more uniform film deposition and enhanced carrier concentration. This was the basis of forming n-PEDOT: PSS layer in the homojunction diode (see section “Device analysis”) using 20 ml/h flow rate.

The pristine (p-type) PEDOT: PSS film was prepared using the drop-casting technique, and a summary of its electrical properties is presented in Table 2. From Table 2, it can be deduced that the carrier concentration is positive, indicating that the prepared film is a p-type material.

**Table 2:** The electrical properties of pristine PEDOT: PSS.

Sample	Carrier type	Conductivity (S/cm)	Resistivity (Ωcm)	Sheet resistance (Ω/sq)	Mobility (cm^2^/(V s))	Carrier concentration (cm^−3^)
PEDOT: PSS	p-type	6.08 × 10^−3^	1.64 × 10^2^	3.31 × 10^3^	25.04	7.58 × 10^13^

### Thermoelectric properties of n-PEDOT: PSS

Thermoelectric (TE) power generation is the transformation of effective heat energy into electricity. The primary factor utilized to access the TE performance of polymers is the power factor (PF). This is due to the difficulty in measuring the heat conductivity of polymers, which is close to zero or low, and does not alter much in response to inorganic doping. In this work, the PF and Seebeck coefficient were determined using the relations^45,46^.

5 $${\text{PF }} = {\text{ S}}^{{\text{2}}} \sigma$$

6 $$S = {-}\frac{V}{{\Delta T}}$$

Where $\:\varDelta\:T$ is in temperature and $\:V$ is the potential difference across the terminals, σ is the conductivity, and S is the Seebeck coefficient. The nature of the charge carriers in n-type and p-type materials causes differences in the Seebeck coefficient in p-type and n-type PEDOT: PSS. The charge carriers in n-type PEDOT: PSS are negatively charged electrons. Electrons migrate from the hot side to the cold side when a temperature gradient is introduced. The Seebeck coefficient is negative because of the accumulation of negative charge on the cold side caused by this electron migration. On the other hand, the charge carriers in p-type PEDOT: PSS are holes, which function as positively charged particles. Holes flow from the hot side to the cold side of a temperature gradient. A positive Seebeck coefficient results from this positive charge accumulation on the cold side. Because of how differently electrons and holes react to temperature gradients, the Seebeck coefficient’s sign and magnitude vary depending on which of the two principal charge carriers is present.

**Fig. 6:**
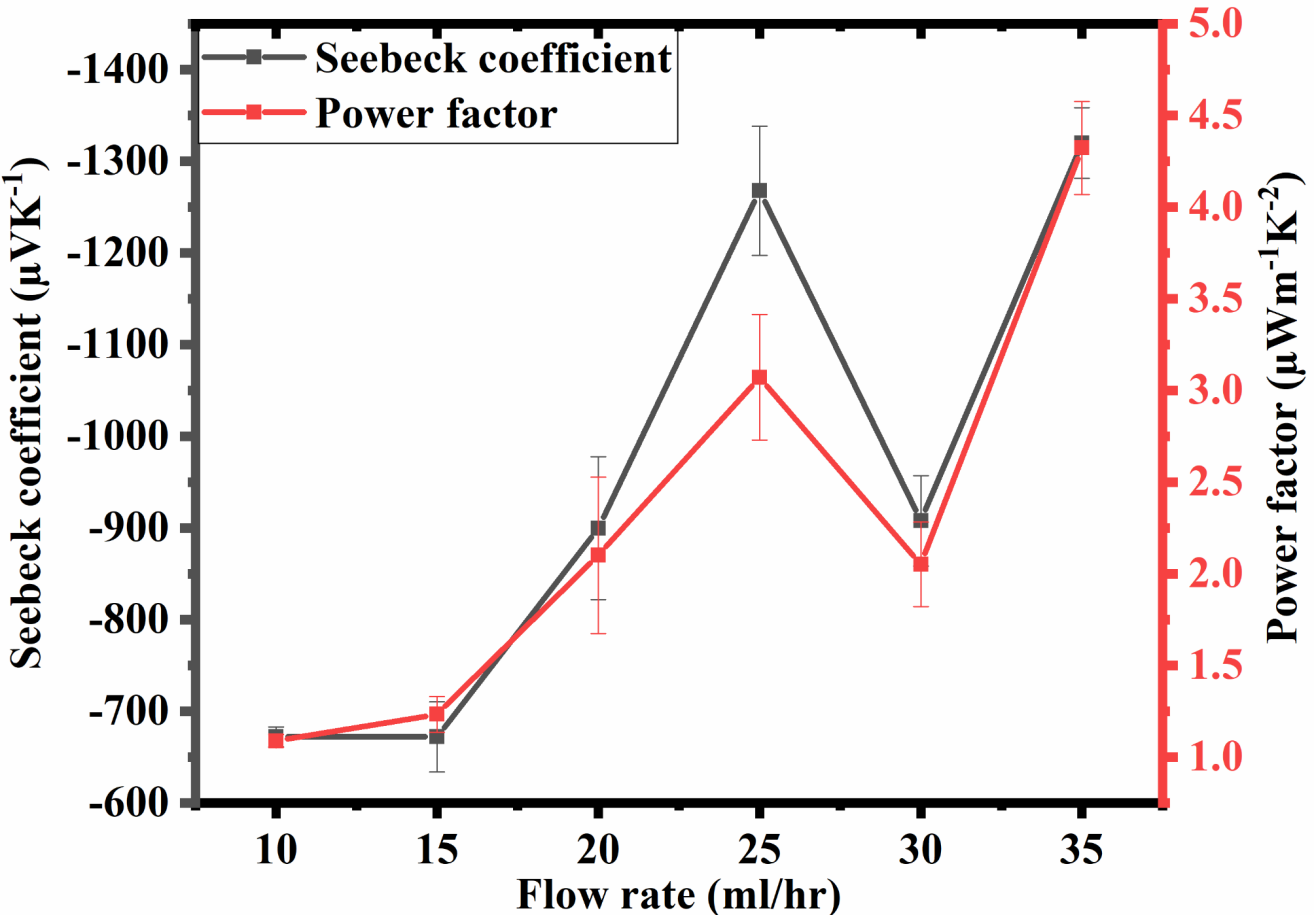
The plot of PF and Seebeck coefficient of n-PEDOT: PSS film.

Figure 6 shows a plot of PF and Seebeck coefficient at various spray flow rates. The values of the Seebeck coefficient and PF increase as the spray flow rate increases. The negative value of the Seebeck coefficient in n-PEDOT: PSS is due to the generated negative charge carriers^47^. The high value of the Seebeck coefficient is very important because PF, which is the main component used in evaluating the TE performance, depends on it. Therefore, high-value PF is recommended as a tactic to increase the output and efficiency of TE power generation^48^. Table 3 compares the PF and Seebeck coefficient of n-PEDOT: PSS with other p-type and n-type polymer composites. It is found that the synthesized n-PEDOT: PSS exhibits excellent TE properties compared with those obtained in the literature.

**Table 3:** The comparison of thermoelectric properties of n-PEDOT: PSS with other polymer composites.

S/*N*	Polymer composite	Seebeck coefficient (µVK^−1^)	Power factor (µWm^−1^K^−2^)	Refs.
1	PEDOT: PSS/BPO_4_	−1360	88.8	^49^
2	PVDF/Bi_2_Se_3_	–90	40.1	^50^
3	PVDF/Ni	–20.6	220	^51^
4	PEDOT: PSS/TiS_2_	–1080	1516	^52^
5	N-DMBI doped PBN-19	−87.09	0.22	^53^
6	PVDF/Cu_0.6_Ni_0.4_	−49.9	189.7	^54^
7	NHC doped PBN-19	−387	5.62	^55^
8	PSDA/PTh	−2535	0.031	^56^
9	PEEK/CNF	−3.4	3.1	^57^
10	PEDOT	−21	0.9	^58^
11	PEDOT: PSS	22.8	122.3	^46^
12	PEDOT: PSS	142	112	^59^
13	Te-PEDOT: PSS	334.68	284	^60^
14	PEDOT: PSS	14.53	1.33	^61^
15	n-PEDOT: PSS	– 1320.06 ± 38.42	4.32 ± 0.25	This work

### Device analysis

#### I–V measurement

**Fig. 7:**
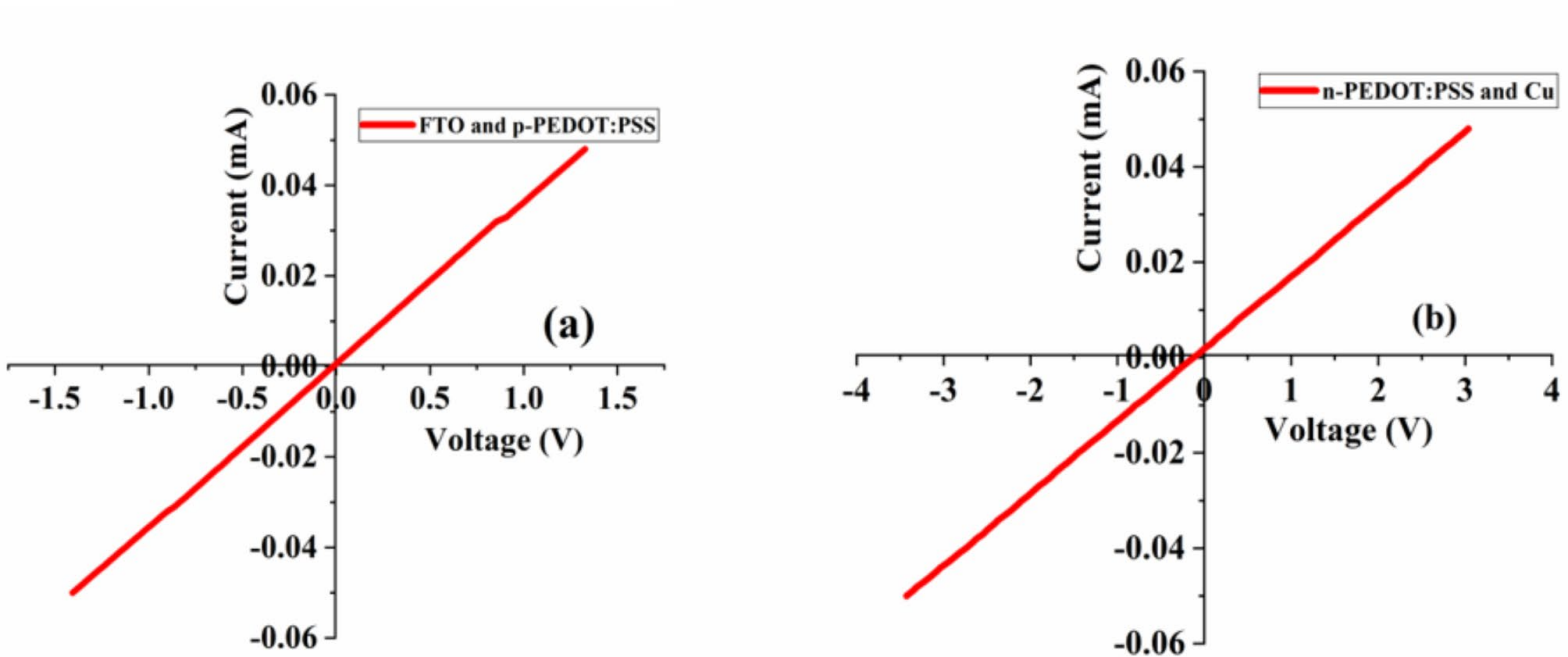
The I-V curve of (a) FTO contact and p-PEDOT: PSS layer and (b) Cu contact and n-PEDOT: PSS layer.

**Fig. 8:**
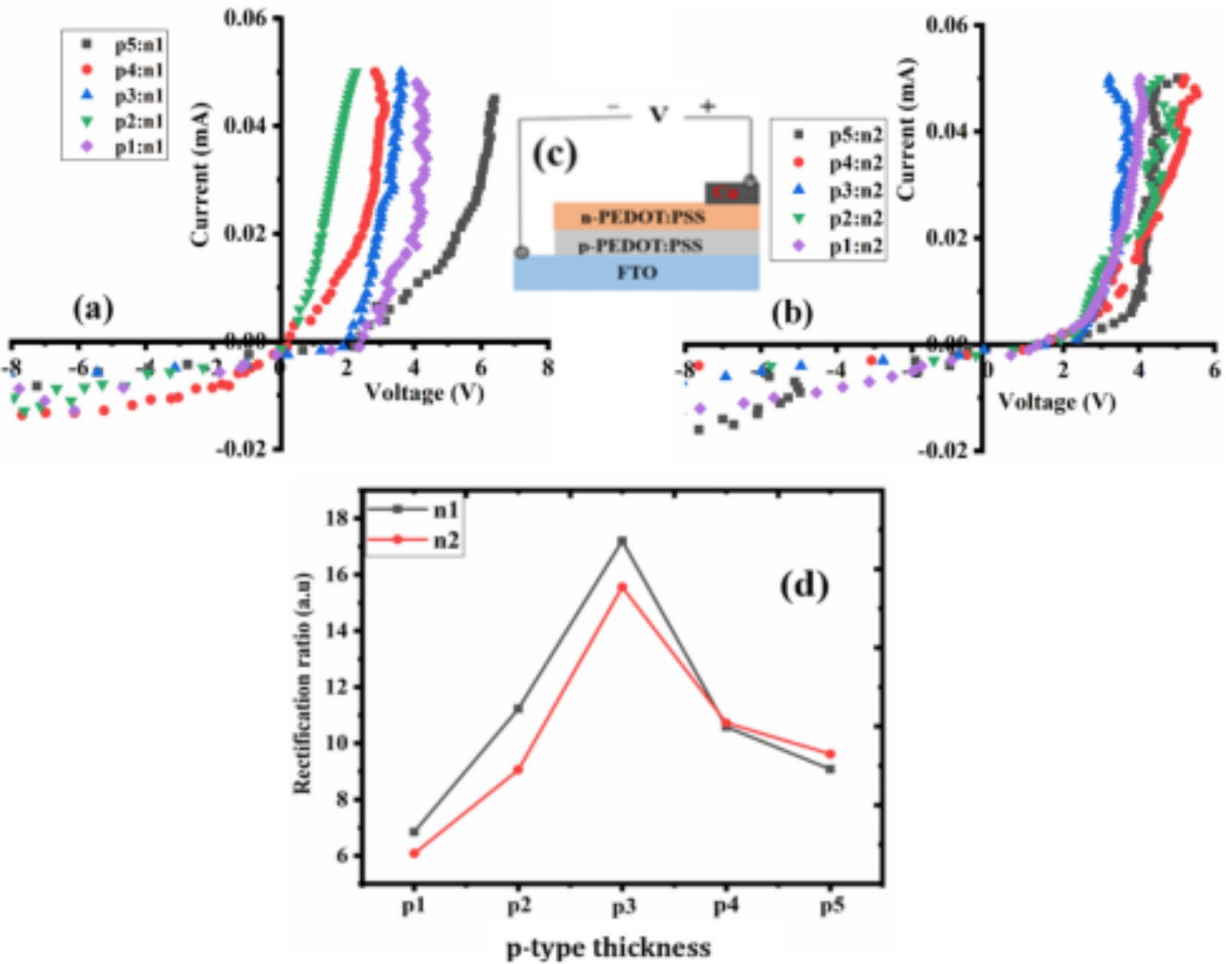
The (a & b) I-V curve of PEDOT: PSS-based homojunction diode device (c) a schematic of the device structure (d) individual *r* of PEDOT: PSS-based homojunction diode for 10 devices with various thicknesses.

The Hall Effect measurement system was used to measure I-V curve of the PEDOT: PSS-based homojunction diode and contacts of the layers. Figure 7 shows the I-V plot between n-PEDOT: PSS layer and Cu electrode contact and p-PEDOT: PSS and FTO contact. It is confirmed that there is an ohmic contact between the n-PEDOT: PSS layer and Cu contact since the I-V curve displays a linear relationship. The p-PEDOT: PSS layer and FTO exhibited comparable features with n-PEDOT: PSS layer and Cu. The I-V curve of the fabricated PEDOT: PSS-based homojunction diodes is shown in Fig. 8a-c for several devices. The curve showed rectification characteristics, and the diodes’ individual rectification ratio (*r*) is shown in Fig. 8d. The *r* of a diode is the ability of a diode to convert alternate current to direct current^62^. The value of *r* was calculated using the relation $\:\frac{{I}_{f}}{{I}_{r}}$ where $\:{I}_{r}$ is the reversed bias current and $\:{I}_{f}$ is the forward bias current.

From Fig. 8d, it was noted that an increase in the thickness of the p-PEDOT: PSS layer causes an increase in *r*. It reaches the maximum for device p3 and declines with a further increase in p-layer thickness. The best-performing diodes have a thicker p-layer compared to the n-layer. The p-layer of diodes should be thicker than the n-layer for a variety of reasons^63,64^, (i) when the diode is forward-biased, it permits effective minority carrier injection. A wider area for the injection of minority carriers is made possible by the thicker p-layer, which improves current flow; (ii) the thickness of the p-layer influences the width of the depletion region in the diode. A larger depletion region aids in controlling the diode’s properties, including the voltage drop and leakage current, thereby enhancing its performance; (iii) a thicker p-layer also influences the voltage breakdown characteristics of the diode. It permits the diode to endure higher reverse bias voltages before breakdown.

The best-performing devices are p3:n1 and p3:n2. The obtained values of *r* are 17.2, 15.56 for p3:n1 and p3:n2, respectively, which is over 500% better than the *r* of PEDOT: PSS-based homojunction diode reported by S. Aboulhadeed et al.^17^. The obtained *r* in this work is as well comparable to other polymer heterojunction diodes. For example, M. Reza et al.^65^ studied the performances of ITO/ PANI/N719/Ag diode. The diode showed Schottky characteristics, and the obtained *r* was 9.2.

#### The device performance of p3:n1 and p3:n2 diodes

**Fig. 9:**
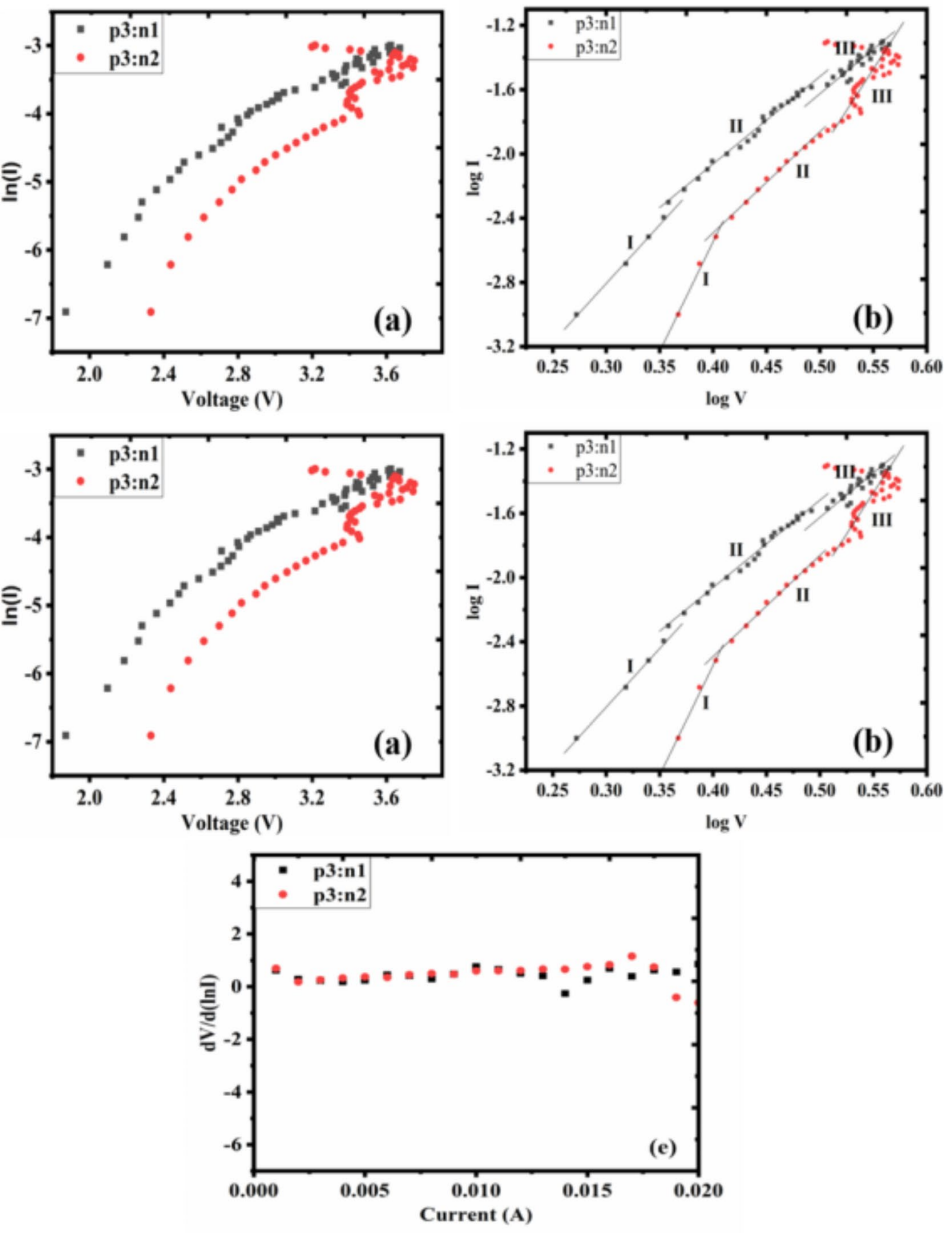
The plot of (a) ln I Vs V (b) log I Vs log V (C) & (d) R Vs V and (e) dV/d(lnI) Vs I of p3:n1 and p3:n2 diodes.

The performance plots of the obtained device (i.e., p3:n1 and p3:n2) are shown in Fig. 9. The barrier height and reverse saturated current were calculated using the relation:^66,67^

7 $${\text{I }} = {\text{ I}}_{0} e^{{ - \frac{{qV}}{{nKT}}}}$$

8 $${\text{I}}_{0} = A^{*} {\text{AT}}^{{\text{2}}} e^{{ - \frac{{\phi _{b} }}{{KT}}}}$$

9 $$\phi _{{\text{b}}} = \left( {\frac{{KT}}{q}} \right){\text{ln}}\left( {{\text{A}}^{*} {\text{A}}\frac{{T^{2} }}{{I_{0} }}} \right)$$

10 $${\text{A}}_{*} = {\text{ 4}}\pi {\text{qm}}*\left( {\frac{{K^{2} }}{{h^{3} }}} \right)$$

Where m^*^ is the effective mass of PEDOT: PSS, A* is the effective Richardson constant, A is the effective mass of a diode, K is the Boltzmann constant, h is the plank’s constant, T is the temperature, ɸ_b_ is the barrier height and I_0_ is the reverse saturation current.

Figure 9a shows the plot of ln(I) against voltage (V). Both p3:n1 and p3:n2 devices exhibited a parabolic curve. The calculated barrier height and reverse saturated current for p3:n1 and p3:n2 diodes are 0.047, 0.059 and 7.88 × 10^−5^ A, 1.11 × 10^−5^ A, respectively. The barrier height is the energy difference between the lowest unoccupied molecular orbital level (LUMO) and the highest occupied molecular orbital level (HOMO), whereas the flow of minority carriers is represented by the reverse saturation current^68^. The ease at which carriers (electrons or holes) pass through the junction is influenced by the barrier height. With 0.047 or 0.059 eV barrier height, carriers should be able to navigate through the junction more easily, allowing for effective carrier movement, this may lead to enhanced diode performance^69^.

The ideality factor (n) was calculated using n =$\:\:\frac{q}{\text{K}\:\times\:\:\text{T}\:\times\:\:\text{S}}$ where S is the slope of ln(I) against the voltage (V) plot. n is a parameter that identifies the degree to which a diode deviates from the ideal diode model in terms of behavior. It is an indicator of the effectiveness and precision of the diode’s operation. p3:n1 and p3:n2 diodes have high n values of 21.3 and 17.1, respectively. The obtained high value of n can be brought on by the presence of high series resistance in the diode circuit, recombination and generation processes within the diode structure and surface defect(s)^70^.

Figure 9b shows the plot of log I versus log V. There are three separate regions on the linear plot corresponding to various diode operation modes. In Region I, the carrier injection serves as the main conduction mechanism. The p3:n1 (0.27–0.35 V) and p3:n1 (0.37–0.41 V) devices display a linear curve, suggesting the diode is not effectively conducting at a lower voltage. Region II of p3:n1 (0.35–0.49 V) and p3:n1 (0.41–0.53 V) show a more pronounced slope region as the voltage rises. The main conduction mechanism in this particular region is a combination of carrier drift and diffusion. An electric field causes the minority carriers—holes in n-type PEDOT: PSS and electrons in p-type PEDOT: PSS—to be dispersed from the junction. Furthermore, some carriers diffuse and pass through the depletion zone. The diode approaches the active or conducting area when there is an increase in current along with voltage. The plot may indicate a saturation or leveling off at very high voltages (in region III), meaning the diode has used up all its available current capacity. The Zener breakdown or avalanche breakdown is the primary conduction mechanism. These mechanisms produce electron-hole pairs quickly because carriers gain enough energy from impact ionization. A similar plot was reported by Mahato^66^ for PEDOT: PSS-based Schottky diode.

Figure 9c and d show the plot of Resistance (Ω) against voltage (V). p3:n1 displays series and shunt resistance of 119.9 and 1872 k Ω, respectively, and p3:n2 has both series and shunt resistance values of 191.9 and 2331 k Ω, respectively. A lower series resistance denotes a higher overall efficiency and lower power loss, and a higher shunt resistance denotes decreased leakage currents and better isolation from parallel routes^71^. Because of its higher shunt resistance, diode p3:n2 may operate better and have fewer negative consequences from leakage current, making it a good contender for some applications.

Figure 9e shows the plot of dV/d(lnI) against I. The graphic aids in identifying the various homojunction diode operating regions. The plot shows a comparatively linear curve when the diode is in the forward-biased. Both p3:n1 and p3:n2 diodes exhibited linear features, and this suggests that the diode behaves as a nearly constant resistance within the operating region^72^.

The properties of p3:n1 and p3:n2 are compared with other diodes in Table 4. It is observed that they show comparable features with Schottky and heterojunction diodes, notwithstanding that they are homojunction diodes. The salient features demonstrated by p3:n1 and p3:n2 envisage that they are unique for the development of next-generation organic electronic devices.

**Table 4:** The comparison of p3:n1 and p3:n2 diodes properties with other diodes.

Diode material	Diode type	*R*_sh_ (KΩ)	*R*_s_ (KΩ)	*n*	Barrier height	Reverse saturation current (A)	Rectification ratio	Ref.
PEDOT: PSS/n-Si	Heterojunction			2.4	0.8			^73^
Glass/ITO/ZnO/PEDOT: PSS/Au	Schottky			1.4	0.9		10^5^	^74^
PEDOT: PSS/p-Si	Heterojunction			1.9	0.70	1.7 × 10^− 7^		^75^
PEDOT: PSS-PVA/n-Si	Heterojunction	0.76 × 10^3^	22.28	4.8	0.81	0.76 × 10^− 6^	2.7	^76^
ITO/PEDOT: PSS/MEH: PPV/Alq3/LiF/Au	Schottky	0.76 × 10^3^	0.31 × 10^3^	20	0.51			^77^
PEDOT: PSS/InGaZnO	Schottky		0.83	1.57	0.77	5.0 × 10^− 7^	1.8 × 10^5^	^78^
Al/ZnO/PEDOT: PSS	Schottky	54.2	49.8	1.3	0.82		1.2 × 10^4^	^79^
Ag/PTCDA/PEDOT: PSS/p-Si	Schottky	137 × 10^3^	13.7	3.5	0.81		236	^71^
PEDOT: PSS/ZnO/n-Si	Heterojunction		2.6	7.8	0.60			^80^
PEDOT: PSS/ZnO	Schottky		0.6 ± 0.2	2.5	0.77	3.5 × 10^− 7^		^81^
p-PEDOT: PSS/ n-PEDOT: PSS	Homojunction	28.9	1.49	14.3	0.013	2.0 × 10^− 5^	3.44	^17,72^
P3:n2	Homojunction	2.3 × 10^3^	191.9	17.1	0.059	1.11 × 10^− 5^	15.56	This work
P3:n1	Homojunction	1.8 × 10^3^	119.9	21.3	0.047	7.88 × 10^− 5^	17.2	This work

## Conclusion

In literature, the reported electrical switching properties of PEDOT: PSS from p-type to n-type have been solely by solvent treatment with isopropanol alcohol (IPA). In this work, we observed that the electrical switching of PEDOT: PSS can also be achieved using spray coating technique. The spray flow rates were varied between 10, 15, 20, 25, 30, and 35 ml/hr. The obtained n-PEDOT: PSS show excellent electrical properties. The conductivity and carrier concentration of n-PEDOT: PSS increases from 1.48 ± 0.03 × 10^−2^ to 2.4 ± 0.06 × 10^−2^ S/cm and – 2.99 ± 0.05 × 10^14^ to – 7.04 ± 0.37 × 10^14^ cm^−3^, respectively, as the flow rate increases from 10 to 35 ml/hr. Furthermore, n-PEDOT: PSS showed a significant Seebeck coefficient of – 1320.06 ± 38.42 µVK^−1^ and a power factor (PF) of 4.32 ± 0.25 µWm^−1^K^−2^. The obtained n-PEDOT: PSS was used to fabricate high-performance PEDOT: PSS homojunction p-n diode and the I-V curve showed high rectification characteristics with a rectification ratio and barrier height of 17.2 and 0.047 eV respectively.

## Data Availability

The data sets used and/or analyzed during the current study are available from the corresponding author upon reasonable request.
